# Convergent Validity of the Cross‐Linguistic Lexical Task

**DOI:** 10.1111/1460-6984.70110

**Published:** 2025-08-11

**Authors:** Svetlana Kapalková, Kamila Polišenská, Andrej Mentel, Tereza Horská, Monika Janíková, Martina Zubáková

**Affiliations:** ^1^ Faculty of Education, Department of Communication Disorders Comenius University in Bratislava Bratislava Slovakia; ^2^ Language and Communication Science City St George's, University of London London UK; ^3^ Division of Psychology, Communication and Human Neuroscience The University of Manchester Manchester UK; ^4^ Faculty of Social and Economic Sciences Institute of Social Anthropology Comenius University in Bratislava Bratislava Slovakia; ^5^ Palacký University Olomouc, Faculty of Arts; Department of Sociology, Andragogy and Cultural Anthropology Olomouc Czech Republic

**Keywords:** convergent validity, cross‐linguistic lexical task, lexical assessment

## Abstract

**Background and Aims:**

The aim of the current study is to assess the validity of the Cross‐linguistic lexical task (CLT) against direct and indirect measures of lexical skills across 2–6 years of age, for a crucial period of lexical development. In addition to evaluating relationships between measures at the level of total score, we also explored agreement at an item level between two lexical measures.

**Method:**

Participants were 109 Slovak‐speaking 2–6‐year‐old typically developing children who all completed the Cross‐linguistic lexical task (CLT‐SK). Three additional measures of lexical skills were obtained: A parental checklist (Slovak adaptation of CDI; *n* = 30, aged 30–36 months), a narrative task to estimate production of Internal State Terms (*n* = 79, aged 41–81 months) and a non‐word repetition task (*n* = 105, aged 30–81). The agreement at the item level was determined between items that were included in both the CLT‐SK and CDI.

**Results:**

Correlational analyses showed that the CLT‐SK was significantly related to all measures. While relationships at the level of total scores were confirmed, agreement at the level of individual items between parental judgement and the child's performance was poor.

**Conclusions:**

The results support the view that CLT‐SK is a valid instrument to assess lexical skills in children aged 2–6 years when total scores are evaluated. However, the agreement at the level of individual items was inadequate, which may have implications for clinical work and/or research based on assumed knowledge of individual items.

**WHAT THIS PAPER ADDS:**

*What is already known on this subject*

- The validity of parental checklists in relation to direct vocabulary assessments is well established, particularly in pre‐school‐aged children. However, there is limited research exploring the relationship between different direct measures of lexical knowledge. This gap highlights the need for further investigation to understand how various methods complement each other in assessing lexical skills.

*What this paper adds to existing knowledge*

- This study supports the convergent validity of the CLT‐SK. It extends current knowledge by exploring relationships between different types of lexical assessments, within a new language, and provides unique insights into the agreement between individual items when evaluated using different methods. The study found that the overall scores of different lexical measures appear to be related, but the validity at the level of individual test items is low.

*What are the potential or actual clinical implications of this work?*

- The results suggest that lexical assessments are effective for evaluating overall vocabulary knowledge but may not reliably measure specific word knowledge. The study supports the validity of the CLT‐SK, highlighting its quick, child‐friendly administration, low costs and high accessibility for diverse populations and clinical settings. These features enhance its utility and contribute to the broader applicability of the CLT framework across multiple languages.

## 1 Introduction

Children's spoken language proficiency is a well‐known predictor of future academic success. Research shows that vocabulary knowledge is essential and that early intervention in lexical development in children who may be experiencing difficulties in their lexicon has been effective. It is therefore important to design tests that accurately assess lexical skills, which will enable the identification of children who might benefit from support. The Royal College of Speech and Language Therapists identified assessment as one of the priority research areas in 2019. Specifically, RCSLT called for the availability of *assessment for DLD, which can track progress: (a) across time, (b) across different environments*. Additionally, also for the availability of *effective tools to assist accurate diagnosis of DLD in early years children*.

The current study focuses on an adaptation of a Cross‐linguistic lexical task (Haman et al. 2015) into Slovak (CLT‐SK). LITMUS CLT‐SK, the Slovak version, was adapted by Kapalková and Slančová (2017) as a paper version and as an electronic version (Sobota et al. 2022). The test assesses naming of concrete objects (nouns) and familiar activities (verbs). The reliability of CLT‐SK task was established in a study by Kapalková and Slančová (2017), but the current study is the first to assess its validity across a range of age groups, from 2‐ to 6‐year‐olds. It is essential to establish if the task provides an accurate estimate of the construct. Importantly, this study also extends the current knowledge on relationships between types of assessments of lexical skills by exploring them in novel combinations, in a new language and by providing insight into agreement between individual items when assessed by different methods. Those areas have been neglected in research, and few studies have examined lexical skills in a variety of ways: directly via assessing a child's performance versus indirectly via parental checklists. The following sections will introduce the CLT task in more detail and discuss studies which addressed the relationship between different types of assessments.

### 1.1 Background and Design of CLT Tasks

The CLT principles were developed within the framework of the COST Action IS0804 Language Impairment in a Multilingual Society: Linguistic Patterns and the Road to Assessment (Haman et al. 2015, also see CLT—Multilada), in order to assess lexical skills in children from 3 to 6 years. The test assesses production and comprehension of concrete objects (nouns) and familiar activities (verbs). There are currently 38 language versions of the CLTs available. While the target words are selected based on a common set of underlying cultural and linguistic principles, the word sets are unique for each language. Those common principles make the lexical test unique for cross‐linguistic research and also for multilingual children, as child's knowledge in multiple languages can be assessed in a comparable manner.

The design of the CLT task is underpinned by a set of principles applied in each language version (see Haman et al. 2015 for the general principles and Slančová & Kapalková, 2013 for Slovak). A number of studies have taken up the CLT task with a wide range of populations, as illustrated by numerous publications. For example, Haman et al. (2017) reported CLT results for monolingual children aged 3; 0–6; 11 across 17 languages. More recently, O'Toole (2024) illustrated the benefit of the CLT task in bilingual Irish–English‐speaking children. Together, those studies illustrate the potential of the CLT task and its uptake across diverse communities.

### 1.2 Parental Checklists and Lexical Knowledge

The relationship between the indirect measures of vocabulary checklists and direct assessment of lexical skills has been evaluated across a wide range of populations and languages. Those studies assess the concurrent validity by evaluating the strength of the associations on total scores between the direct and indirect assessments, generally reporting significant moderate correlations (e.g., Czech: Smolík and Bytešníková 2021, *r =* 0.53 for production, *r* = 0.39 for comprehension; English: Feldman et al. 2005: *r* = 0.41; Estonian: Tulviste and Schults 2023 *r*  =  0.64–0.69; Maltese: Gatt et al. 2023 *r* = 0.63).

The majority of studies evaluating the relationship between tools report correlations. However, if a test establishes validity on the basis of the total scores, it may not mean that the assessment of individual items is also reliable. Bennetts et al. (2016) highlighted the issue while looking at correlations between total scores on direct and indirect measures and demonstrated that while the strength of the relationship between two measures can be estimated by looking at correlations, this is different to agreement between the measures. Within the language development field, not many studies addressed this issue. One early exception was a study by Ring and Fenson (2000) with 40 English‐speaking children aged 19–31 months while assessing their lexical knowledge of the same items by different means. The children were administered the Receptive Vocabulary Task (35 items) and Expressive Vocabulary task (29 items), and a MacArthur CDI Words and Sentences forms. The items in the expressive and receptive tasks did not overlap; they were selected to be representative across the age range and varied in the level of difficulty. The study reported that there was an overall correlation between total comprehension scores on the direct assessment and CDI receptive scores (*r* = 0.55) and similarly between expressive scores and CDI expressive score (*r* = 0.67). Although Ring and Fenson (2000) considered performance on the same items, their analyses were performed on the mean proportion accuracy scores either for totals for comprehension/production or across items by level of difficulty. They reported that parents showed wider distribution of scores compared to children's performance, and their scores were higher compared to children. This could be because parents overestimated their children's knowledge and/or children underperformed in the direct assessment, for example, due to task demands or unfamiliarity of being tested.

More recently, Łuniewska et al. (2024) assessed the lexical knowledge of identical items via two methods as follows: direct assessment of lexical items by naming or picture selection and indirectly by parental reporting, that is, indirect assessment of the same selection of items. Their study found that while the overall direct and indirect scores significantly correlated (production *r* = 0.36 and comprehension *r* = 0.33), parents rated children's knowledge significantly higher for comprehension (direct measure < indirect measure), but there was no significant difference for production (direct measure = indirect measure). When the knowledge was assessed at the participant level, the agreement was poor. Similarly, agreement at the item level was found to be low to moderate and was related to the level of vocabulary and age, with poorer agreement for older children with larger vocabulary. Although the sample of 94 children was large, the age ranges included might have been less suitable for the parental checklist method (3–6 year olds, median age 4.88). Bennetts et al. (2016) found that agreement was generally stronger for children at the ends of the distribution, that is, with either poorer or exceptional language abilities, while agreement for children with average language abilities was weaker.

### 1.3 Non‐Word Repetition (NWR) and Lexical Knowledge

There has been an ongoing theoretical debate about the relationship between NWR and vocabulary knowledge (e.g., Gathercole 2006). The link between the two skills is well established and demonstrated across many languages and ages, with children who perform well on NWR also scoring higher in vocabulary tasks (Hoff et al. 2008; Polišenská and Kapalková 2014). However, the nature and the developmental trajectory of the relationship are less clear (Melby‐Lervåg et al. 2012; Willard et al. 2021). Significant correlations have been reported between direct and indirect measures of vocabulary and NWR (e.g., Jones 2016; Polišenská and Kapalková 2014). Although NWR is often conceptualised as a measure of phonological working memory, the items used in our study were highly wordlike and contained familiar morphemes. This increases reliance on long‐term lexical and morphological knowledge in addition to phonological processing. Indeed, previous studies have demonstrated that NWR performance is influenced by factors stored in long‐term memory, such as phonotactic probability and wordlikeness (Gathercole 2006), which strengthen the expected association between NWR and broader language knowledge. In sum, the relationship between vocabulary size and NWR performance appears to hold across a number of variables, such as the language assessed, the type of NWR task, the ages and vocabulary size of the children tested and the type of assessment used for evaluation of lexical skills.

### 1.4 Internal State Terms (ISTs) and Lexical Knowledge

As children grow older, lexical items related to abstract concepts such as inner experience, emotions or cognitive concepts increase. However, most direct and indirect assessments primarily assess the production of concrete nouns and verbs. ISTs are primarily expressed through metalinguistic, metacognitive, motivational and perceptual verbs and emotional adjectives (see ‘Materials’ for examples). While ISTs are traditionally viewed as a measure of Macrostructure in children's narrative, a number of studies have argued that ISTs could also be seen as a measure of lexical knowledge (e.g., Fichman et al. 2022; Chan et al. 2023).

The developmental trajectory of ISTs has been documented across a number of studies (e.g., Welliver et al. 2024). However, there is limited research into how ISTs relate to general lexical skills. As most lexical assessments utilise pictures as a means for eliciting response (either naming or selection from pictures/objects), ISTs are unlikely to be included as most of them are not readily suited due to their low imageability. They are, nevertheless, an important part of lexical knowledge and as research shows, children start adding them to their dictionary from the age of 2 and accelerating in their third year (Furrow et al. 1992). Their evaluation is also important clinically. For example, a study by Boerma et al. (2016) found that children with DLD produced fewer ISTs compared to TD children.

Most studies which considered the relationship between ISTs and general vocabulary in younger children used parental reports as a source of information about ISTs’ knowledge. Roberts et al. (2021) have shown a significant positive relationship (*ρ* = 0.825) between the number of mental state terms and total vocabulary as assessed by CDI in English‐speaking 24‐month‐old children. Similarly, Chiarella et al. (2013) found children's expressive vocabulary skills to be significantly related to Internal‐State Checklist scores (*r* = 0.34, *p* < 0.01) in a sample of 75 German‐speaking 30‐month‐olds. Relatedly, correlations have been shown between direct standardised general vocabulary measures and more specific groups of vocabulary items such as receptive emotion vocabulary in older children (Sturrock and Freed 2023). Taken together, knowledge of ISTs has been shown to be related to general vocabulary skills, and limited studies with children with language disorder indicate that ISTs might be particularly vulnerable lexical items, compared, for example, to more general vocabulary; therefore, assessing them might provide valuable insight and relevant information for clinicians. There is some indication that ISTs relate to general vocabulary in young children; however, the relationship is not well‐studied in pre‐school and school age children.

### 1.5 Current Study

The aim of the current study was to evaluate the convergent validity of the Slovak version of the CLT‐SK. A range of tasks known to assess lexical skills across various ages was administered, and their scores were assessed against the CLT‐SK. This is a first study to evaluate the validity of this task in Slovak. Although carried out in Slovak, the study goes beyond a specific language by investigating the validity of lexical tasks more broadly by employing a range of direct and indirect assessment tasks and by considering the validity of the task as a whole (total score) as well as at an individual level. Surprisingly, research addressing this area is limited, even though findings would have important methodological as well as clinical implications. Therefore, this study presents a novel contribution to this debate. Specifically, we address the following research questions:

RQ1: What is the relationship between vocabulary scores derived via parental checklist and CLT‐SK?
RQ2: What is the agreement at the level of individual items as assessed via CLT‐SK vs parental checklist?

The two methods differ in how they assess vocabulary: namely, direct versus indirect methods (CLT‐SK vs. CDI). Based on previous research, we expect to see a relationship between the total scores of direct and indirect measures. The previous findings on agreement at the individual level have been limited, but indicating poor agreement. We expect the agreement to vary depending on the level of difficulty of the items, with early acquired items reaching good levels of agreement, while others showing acceptable or poor levels of agreement.

RQ3: What is the relationship between CLT‐SK and NWR scores?

Given numerous previous reports of a significant relationship between NWR scores and vocabulary assessments, we expect to find a significant relationship between those two measures in a sample of 2–6‐year‐old Slovak‐speaking children.

RQ4: What is the relationship between CLT‐SK and ISTs obtained via narrative?

We hypothesised that general vocabulary (CLT‐SK) would be associated with the number of ISTs in children's narrative, such that children with higher expressive vocabulary will produce more different ISTs.

The unique contribution of our study is in evaluating lexical skills across different ages and methods. It is also the first study to systematically evaluate lexical skills in Slovak‐speaking children, with the focus on the CLT‐SK measure and its relationships to other methods known to assess vocabulary knowledge. It is important to establish how the methods relate to each other and consider their limitations. This understanding can then guide clinicians on how and when to use the methods and what information the methods can and cannot provide.

## 2 Method

### 2.1 Participants

The study received an ethical approval from the Research Evaluation Ethics Committee at Faculty of Education Comenius University in Bratislava for a project entitled: Vocabulary as an indicator of the developmental language level of monolingual and bilingual children in preschool age, APVV 20‐0126. The validity of CLT‐SK in this study was assessed in a sample of 109 children. Recruitment was targeted via various groups such as parental groups, churches and nurseries. Generally, the parents who took part could be described as middle class, predominantly with middle/university education. Parents/guardians were asked to fill in a background questionnaire about the general development of the children. No parents expressed concerns about their child's development, and no children from our sample were referred to speech and language therapy services for language concerns. In addition, all children were seen by a paediatrician as part of a health check. This health screening is compulsory in Slovakia. During the visit, children are assessed in terms of their general motoric, cognitive and social development, and parents are asked to provide information through a general standardised questionnaire (http://www.zdraviedietata.sk/). None of the children in our sample was identified as at‐risk. In summary, neither parents/guardians nor paediatricians expressed any concerns; therefore, we considered the children in our sample to be typically developing. Informed consent was obtained from all participants.

All 109 children were administered the CLT‐SK task, but due to the design and suitability of the assessments for certain ages, not all children could be given all the assessments described in ‘Materials’. The study included two participant samples; for further details on the sample and tasks used, see Table 1. Only the younger group received the CDI task, as the CDI is not suitable for children aged above 3 years, while only the older group completed MAIN, since MAIN is not suitable for children below 3 years of age (Gagarina et al. 2019). All children received CLT‐SK and NWR as both tasks were suitable for all ages included in this study.

**TABLE 1:** Descriptive information about the participants’ samples.

Sample	Number	Gender F/M	Age range	Mean age (SD)	Tasks	Relevant RQ(s)
Sample 1	30	17/13	30–36	33.07 (2.15)	CLT‐SK CDI NWR	RQ1 RQ2 RQ3
Sample 2	79	43/36	41–81	59.41 (9.73)	CLT‐SK NWR ISTs	RQ3 RQ4

Note: Samples 1 and 2 were used jointly to address RQ3 (*N* = 30 + 79 children, CLT‐SK and NWR).

### 2.2 Materials

**CDI Short version TEKOS II** (Kapalková and Kaletová 2020). The task has 81 items and for each item, parents/guardians indicate if their child ‘says’/‘understands’/‘does not say nor understand’. The short version of the TEKOS‐II (CDI) was developed for children aged 17–36 months. Validity was measured between long and short versions of the Slovak standardised CDI—TEKOS II (*r* = 0.963 production). Test–retest reliability measured 14 days apart showed strong correlation for production (*r* = 0.870).

**NWR task** described in detail in Polišenská and Kapalková (2014). The task consisted of 26 items varying in phonological complexity and syllable length. The stimuli were recorded and administered individually over a laptop computer; the administration took approximately 5 min. Whole‐item scoring from recordings was used, and items were scored as correct if all phonemes were produced in the correct order; any omissions, substitutions or additions resulted in an incorrect answer. The maximum score was 26. The inter‐rater reliability was assessed via Pearson correlations of the raw score for two raters, based on 20 recordings: *r* = 0.951, suggesting excellent reliability.

**Cross‐lexical task (CLT‐SK)**. The current study only used the production part (‘naming’) of the CLT‐SK, with a maximum score of 64, with 32 nouns and 32 verbs. All targets were elicited by picture stimuli (coloured drawings) designed specifically for the CLT. Children's responses were recorded, transcribed and scored as correct or incorrect. For a detailed list of acceptable responses, see Kapalková and Slančová (2017, Tables 1 and 2 in the Appendix). Kapalková and Slančová (2017) reported reliability of the CLT‐SK task as follows: internal consistency—Cronbach *α* = 0.899, test−retest reliability based on 22 children tested within 2 weeks: *r* = 0.934.

**Internal state terms (ISTs)**. The number of different ISTs was obtained from a sample of children's narratives elicited by the MAIN task (Gagarina et al. 2019; Kapalková et al. 2020). The MAIN was developed within the framework of the COST Action IS0804 in order to assess narrative skills in children aged 3–10 years. The current study used the production part, specifically, children were asked to tell one of two stories, either Baby Goats or Baby Birds. The narrative was transcribed by a qualified speech and language therapist with experience in transcription using the CHAT format (MacWhinney 2000). The type count was each type of the ISTs produced according to the MAIN manual, with a manual check to correct for any morphophonological changes that could affect the count. The ISTs included the following: perceptual state terms (e.g., *vidieť* [see]), physiological state terms (e.g., *únava* [tiredness]), consciousness terms (e.g., *ospalý* [sleepy]), emotion terms (e.g., *znepokojený* [worried]), cognitive verbs (e.g., *zabudnúť* [forget]) and linguistic verbs (e.g., *povedať/hovoriť* [say/tell]). Two raters independently transcribed and coded 20 children (20/79 = 25%); tokens, ICC = 0.873 (95% CI: 0.711–0.948;); types, ICC = 0.837 (95% CI: 0.632–0.932). The inter‐rater reliability was high.

### 2.3 Procedure

Each child was assessed individually. For Sample 1, parents/guardians were sent the CDI form in advance and asked to bring it completed to the testing session where CLT‐SK was administered. The time between completing CDI and CLT‐SK was a maximum of 1 week. For Sample 2, children between 30 and 35 months were given the CLT‐SK task first, followed by the NWR task. The session lasted a maximum of 30 min. Children who were 36 months and older were also administered the MAIN task. The order of the tasks was the same for all children: CLT‐SK, MAIN and NWR. The session lasted a maximum of 1 h. Four children were excluded as they did not complete the NWR task, leaving the final sample to address RQ3 as *N* = 105 (Samples 1 and 2).

### 2.4 Analysis Plan

First, we assessed the relationship among CLT‐SK scores and other lexical tasks (CDI, NWR and ISTs) via Pearson's correlations. Furthermore, to address RQs 1, 3 and 4, Poisson regression with a logarithmic link function was used (Hilbe 2011), as the outcome variables represented count data (i.e., the number of correctly produced words, repeated non‐words or ISTs). Poisson regression is appropriate for modelling discrete, non‐negative outcomes that follow a count distribution, particularly when the variance is expected to be proportional to the mean. This approach was chosen because all dependent variables were counts of correct responses bounded at zero, and preliminary data inspection showed no substantial overdispersion that would necessitate the use of a negative binomial regression. Poisson regression was therefore considered appropriate. Within this framework, different sets of predictors and covariates were compared using Akaike and Bayesian information criteria to identify the best‐fitting model specification for each outcome (see Table 3). We also analysed the agreement between the direct and indirect vocabulary measures on seven items that were present in two tasks. We evaluated item‐level agreement between parental reports and child accuracy by computing Cohen's Kappa for each item. All calculations were performed in the R statistical environment (R Core Team 2023); graphical outputs were produced using the ggplot2 (Wickham 2016) functions within the R environment.

## 3 Results

### 3.1 Correlations

As can be seen from Table 2, CLT‐SK correlated positively and significantly with all three lexical metrics (CDI, NWR and ISTs).

**TABLE 2:** Descriptive statistics and Pearson correlations for study variables.

Variable	*N*	*M*	SD	1	2	3	4
1. CDI	30	70.47	10.99	1			
2. ISTs	79	1.89	1.83	.^a^	1		
3. NWR	105	13.10	5.40	0.279	0.164	1	
4. CLT‐SK	109	40.27	11.33	0.631^**^	0.323^**^	0.457^**^	1

Notes:

^a^ Cannot be computed because at least one of the variables is constant or not available in the data set.

** *p* < 0.01.

Next, we used Poisson regression, controlling for age and gender. In all cases, the lowest values of AIC and BIC are obtained for models containing controlling variables (age and gender) as well as the predictor (i.e., CLT‐SK score), see Table 3.

**TABLE 3:** Model comparison using Akaike and Bayesian information criteria (AIC and BIC).

Model	Outcome	Predictors	*df*	AIC	BIC
m0.1	CDI	Intercept	1	238.47	239.87
m1.1	CDI	Age + gender	3	226.13	230.34
m2.1	CDI	Age + gender + CLT‐SK	4	219.68	225.29
m0.3	NWR	Intercept	1	712.68	715.33
m1.3	NWR	Age + gender	3	692.47	700.44
m2.3	NWR	Age + gender + CLT‐SK	4	667.64	678.26
m0.4	ISTs	Intercept	1	290.18	292.55
m1.4	ISTs	Age + gender	3	277.33	284.43
m2.4	ISTs	Age + gender + CLT‐SK	4	272.37	281.85

### 3.2 Indirect Assessment of Vocabulary via CDI as a Function of CLT‐SK Vocabulary Score

**FIGURE 1:**
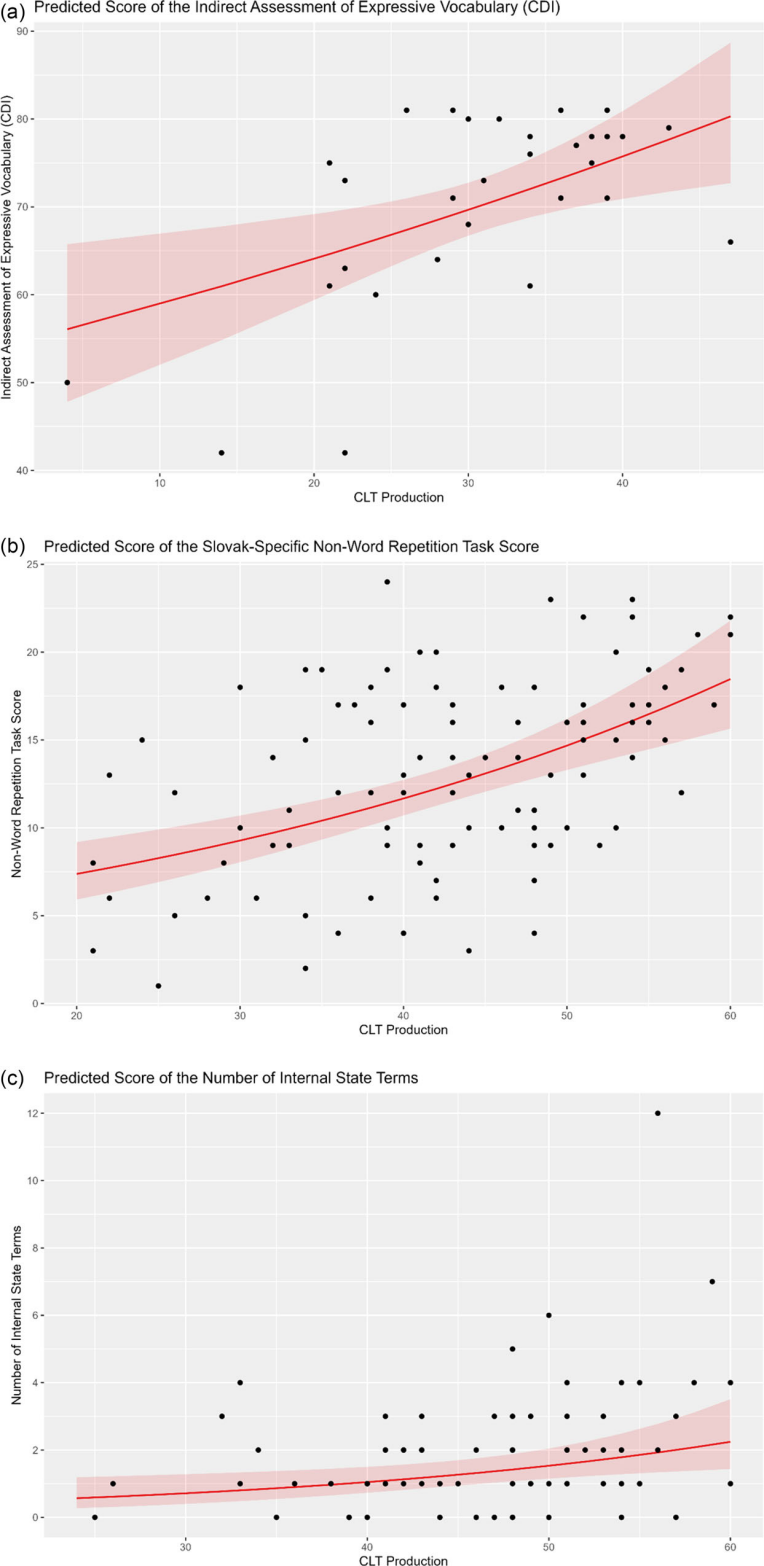
(a–c) Plots of the main effects as a function of CLT‐SK vocabulary scores.

The CDI score of the indirect assessment of vocabulary (the outcome variable) is the number of words productively used by the examinee. According to the Poisson regression results, while controlling age and gender, a one‐unit (one word) increase in CLT‐SK vocabulary score was associated with a 0.8% average increase in the CDI score (see Table 4 and Figure 1). The model yielded a Nagelkerke's Pseudo‐*R* ^2^ of 0.673; the McFadden's Pseudo‐*R* ^2^ of 0.71.

**TABLE 4:** Poisson regression: overview of lexical scores as a function of CLT‐SK vocabulary score.

Indirect assessment of expressive vocabulary (CDI)						
Predictors	Estimate (*β*)	SE (*β*)	*z*	Incidence rate ratios	CI	*p*
(Intercept)	3.194	0.372	8.594	24.39	11.75–50.43	**<0.001**
Age	0.023	0.012	1.972	1.02	1.00–1.05	**0.049**
Gender‐F	0.067	0.050	1.359	1.07	0.97–1.18	0.174
CLT‐SK	0.008	0.003	2.896	1.01	1.00–1.01	**0.004**

Slovak‐specific non‐word repetition task						
(Intercept)	1.657	0.138	11.990	5.24	3.99–6.86	**<0.001**
Age	−0.002	0.003	−0.768	1.00	0.99–1.00	0.443
Gende‐ F	0.050	0.055	0.918	1.05	0.94–1.17	0.358
CLT‐SK	0.023	0.004	5.157	1.02	1.01–1.03	**<0.001**
Internal state terms						
(Intercept)	−1.815	0.638	−2.844	0.16	0.05–0.55	**0.004**
Age	0.006	0.011	0.524	1.01	0.98–1.03	0.600
Gender‐F	0.482	0.176	2.737	1.62	1.15–2.30	**0.006**
CLT‐SK	0.038	0.015	2.606	1.04	1.01–1.07	**0.009**

Note: The bold font indicates statistical significance (*p* < 0.05)

### 3.3 Agreement at the Level of Individual Items: CLT‐SK and Parental Checklist (CDI)

The agreement was analysed at the level of individual items rather than at the level of respondents, as the aim was to examine how specific lexical items behave across two different assessment modalities, one based on the child's performance (CLT‐SK) and the other on parental report (CDI), rather than to evaluate individual parental response patterns. Agreement between the two sources was therefore assessed from the perspective of items (i.e., vocabulary entries shared across both tools), rather than from the perspective of evaluators (i.e., parents). This analysis was conducted on a subsample of 30 child–parent dyads, focusing on the seven lexical items that were present in both the CDI and the CLT‐SK. Table 5 describes the confusion matrices for each of the relevant seven items presented in both instruments. In these matrices, each row represents the actual response category based on the parental checklist (CDI), while each column corresponds to the predicted response based on the child's performance in the CLT‐SK. For this analysis, only expressive vocabulary responses were included: items marked as ‘says’ on the CDI were coded as *positive*, while items marked as either ‘understands’ or ‘does not say nor understand’ were coded as *negative*. Similarly, in the CLT‐SK assessment, a correct naming of an item by the child was coded as *positive*, and an incorrect or missing response was coded as *negative*. Each confusion matrix (Table 5) displays the agreement for a specific item across the two assessment methods, showing the number of cases with correct knowledge in both sources (true positives), incorrect knowledge in both (true negatives) and the two types of disagreement (false positives and false negatives). The diagonal of each matrix represents the cases of agreement.

**TABLE 5:** Confusion matrices for selected items.

Actual condition: CDI		Predicted condition: CLT‐SK	
		Positive	Negative
Ananás (a pineapple)			
	Positive	10	12
	Negative	1	7
Auto (a car)			
	Positive	30	0
	Negative	0	0
Pomaranč (an orange)			
	Positive	12	14
	Negative	0	4
Piť (to drink)			
	Positive	28	2
	Negative	0	0
Plakať (to cry)			
	Positive	27	3
	Negative	0	0
Voňať (to smell)			
	Positive	11	14
	Negative	1	4
Lepí–lepidlo (to glue/a glue)			
	Positive	6	18
	Negative	0	6

For three items, Cohen's kappa is either impossible to calculate or equal to 0, because one or both variables were constant. For the item *auto* (a car), this means perfect agreement between the direct and indirect assessment. In the further two cases, *piť* (drink) and *plakať* (cry), CDI shows no negative cases. Here, children responded mostly correctly, so the true positives vastly outnumbered the false positives. In the remaining four cases, the agreement between direct and indirect assessment is none to slight (0.01–0.20) for three items and fair (0.21–0.40) for one item: (*ananás* (a pineapple) *κ* = 0.229, SE = 0.126; *pomaranč* (an orange) *κ* = 0.186, SE = 0.092, *voňať* (to smell) *κ* = 0.118, SE = 0.11; *lepí–lepidlo* (to glue/a glue) *κ* = 0.118), SE = 0.026.

### 3.4 NWR Task Score as a Function of CLT‐SK Score

The NWR task score was assessed together with CLT‐SK in a sample of *n* = 105; *n_females_* = 58; age: 30–81 months, *M* = 52.90, SD = 14.20. According to the Poisson regression results (see Table 4 and Figure 1b), controlled for age and gender, a one‐unit (one word) increase in CLT‐SK score was associated with a 2.3% average increase in the NWR score.The model yielded a Nagelkerke's Pseudo‐*R* ^2^ of 0.421, the McFadden's Pseudo‐*R* ^2^ of 0.062.

### 3.5 ISTs as a Function of CLT‐SK Vocabulary Score

The outcome variable in this case was the number of ISTs produced by the examinee in the narrative sample (MAIN task) obtained from 79 children; *n_females_* = 43; age: 41–81 months, *M* = 59.41, SD = 9.73. According to the Poisson regression results (see Table 4 and Figure 1c), while controlling for age and gender, a one‐unit (one word) increase in CLT‐SK score was associated with a 3.9% average increase in the number of ISTs. The model yielded a Nagelkerke's Pseudo‐*R* ^2^ of 0.334 and the McFadden's Pseudo‐*R* ^2^ of 0.055.

## 4 Discussion

Our study evaluated the convergent validity of a vocabulary CLT‐SK task and the relationships between different measures of lexical knowledge. The study assessed children's expressive lexical skills via multiple assessment methods across the ages of 30–81 months in a sample of 109 children. It included four ways of assessing vocabulary that have all been shown to reflect some aspect of lexical knowledge and revealed significant positive relationships between both direct and indirect vocabulary assessments. The findings in the current study thus provide evidence of convergent validity for CLT‐SK.

Similar to previous studies on lexical measures, we found that Pearson correlations between CLT‐SK and other assessment tools showed weak‐to‐moderate relationships. The strongest relationship was observed between direct and indirect assessment of vocabulary (*r* = 0.63), echoing previous studies on CDI adaptations and direct measures in a range of languages as follows: Czech (Smolík and Bytešníková 2021, *r* = 0.53), English (Feldman et al. 2005: *r* = 0.41) and Maltese (Gatt et al. 2023, *r* = 0.63). The relationship also held after controlling for age and gender (with age being marginally significant *p* = 0.049). Our findings support the recommendation of combining direct and indirect assessments of children's vocabulary in early years.

A moderate relationship (*r* = 0.46) appeared between CLT‐SK and NWR, the two direct methods of probing lexical skills. This extends a finding of Polišenská and Kapalková (2014), which reported a significant moderate correlation between Slovak language‐specific NWR scores and vocabulary skills as measured by a parental checklist (*r* = 0.72). Polišenská and Kapalková (2014) established the relationship in fourteen 2‐year‐old children (mean age of 28 months, SD = 2.86), while the current study includes a sample of 105 children aged 30–81 months and it evaluates the relationship between two direct methods (CLT‐SK and NWR). Together, this highlights the common resources children draw on when repeating non‐words and naming nouns and activities. It also extends the age window when this relationship is present and replicates it on a larger sample. In short, the relationship between expressive lexical skills and NWR highlights the common background relevant for children's lexical knowledge for both tasks as assessed across a wide age range and regardless of whether the measure is direct or indirect. While our study was not designed to investigate the nature and directionality of the relationship between NWR and lexical skills, it contributes to the evidence that children with stronger phonological memory as measured by NWR, also show larger vocabulary. However, as our study was cross‐sectional rather than longitudinal, we cannot investigate whether better retention and recall of new words facilitates faster vocabulary growth or if a larger vocabulary supports better recall. Alternatively, the relationship could be bidirectional (e.g., Melby‐Lervåg et al. 2012; Willard et al. 2021).

Our study shows a relatively weak relationship between the other two direct methods of lexical assessment (*r* = 0.32), namely CLT‐SK and ISTs. Nevertheless, it appears that even though the IST's vocabulary is more abstract, children with a larger concrete, highly imageable and depictable vocabulary also have a higher number of ISTs. The relatively weak relationship could reflect differences in how concrete and abstract vocabularies are stored and accessed in the mental lexicon. While both types of vocabulary may share connections, they likely involve different cognitive processes for retrieval and use, leading to the observed modest relationship. Additional explanation could be the distinction between macrostructural and microstructural knowledge. ISTs have been linked to macrostructural components, which encompass the broader organizational aspects of language, such as the ability to express internal states, emotions and mental states. In contrast, CLT‐SK primarily measures microstructural elements, such as concrete vocabulary size and word knowledge. The modest correlation between these two assessments may arise because ISTs reflect broader, more abstract narrative abilities that are only partially dependent on specific vocabulary knowledge. Furthermore, ISTs may also reflect children's developing Theory of Mind abilities, placing them at the intersection of lexical knowledge and socio‐cognitive skills (Muszyńska et al. 2024). This unique positioning can be seen as an advantage, as ISTs tap into a distinctive area of vocabulary use that bridges language and socio‐cognitive development. Nonetheless, complementing IST‐based validation with additional narrative‐derived measures, such as Number of Different Words (NDW), may provide a more comprehensive picture of lexical diversity.

Age was not found to have a main impact in this relationship, but gender was identified as a significant factor, showing that girls tended to express more ISTs compared to boys when general lexical skills, as measured by CLT‐SK, were equal. In other words, on average, a girl with the same CLT‐SK score as a boy would produce a higher number of ISTs. Regarding gender differences in the use of ISTs, previous research appears to be mixed. While some studies found gender differences in favour of girls, others reported a lack of gender differences. For example, Cervantes and Callanan (1998) found differences in 2‐year‐olds, but those differences disappeared at the ages of three and four. Hughes et al. (2007) showed that overall, girls displayed more frequent emotion state talk than boys (sample age range 3.5–4.8 years). In contrast, other studies found no gender differences (e.g., Kristen et al. 2014). It is likely that differences in age, data collection and scoring, sample sizes and the definition of ISTs contributed to the mixed pattern of results across studies. ISTs might also be related to the theoretical view of language development as context‐dependent and emerging from repeated use in meaningful interactions (Tomasello 2003). Considering the usage‐based theories, as ISTs are more abstract and relate to mental and emotional states, they may require richer social contexts for their acquisition. Girls, who are often encouraged to discuss emotions and internal states more frequently, may therefore develop a stronger ability to use ISTs. This context‐dependent learning aligns with the patterns of gender differences observed in IST production in our study.

While our study reported significant correlations, similar in strength to previous studies, the correlations are not strong. Small‐to‐moderate correlations between different measures of lexical skills in children may be observed due to several factors. Different measures often tap into various dimensions of lexical skills, such as word form retrieval, the ability to use words in context or different areas of lexicon (e.g., concrete vs. abstract, words of different imageability). These measures may not always align closely because they assess distinct aspects of language development. Measures based on different theoretical perspectives might capture distinct elements of lexical knowledge, such as depth (understanding word meanings and relationships) versus breadth (the number of words known) or focus more on word meaning versus word form. These differences can result in discrepancies across measures, as they may not fully overlap in what they are intended to assess. Additionally, the age of the children being studied and their stage of language development can influence these correlations. Children's performance can also vary depending on the testing environment, their familiarity with the test format or the test situation itself, or even fluctuations in, for example, children's energy levels or attention span. As a result, these variations may lead to only weak‐to‐moderate correlations across different measures, reflecting the multifaceted nature of lexical knowledge and development in children.

We believe that the findings of mutual relationships confirmed in our study are clinically informative as evaluating a child's lexicon from various angles is meaningful and provides a fuller picture of a child's capabilities in different contexts. Indeed, while a similar construct is being assessed by those various methods, the assessment methods seem to complement each other rather than being mutually exclusive. Numerous studies, including the results of the current study, suggest that the relationship between direct and indirect methods of lexical knowledge is well established and holds across different ages. However, as highlighted by the study of Ring and Fenson (2000) and a recent study by Łuniewska et al. (2024), few studies look at agreement at the level of specific items—words. The rare exceptions of the studies mentioned above highlighted a poor level of agreement. Ring and Fenson (2000), in evaluating the production skills of individual specific lexical items by parents and directly by examining the child, reported an overestimation of children's language ability by parents. Recently, Łuniewska et al. (2024) demonstrated that parents tend to rate children's knowledge significantly higher for comprehension, but not for production. Ring and Fenson's findings were based on 40 English‐speaking children in the age range of 19.8 months to 31.1 months (*M* = 25.45), responding to 35 items for production. Łuniewska et al. (2024) reported results based on a sample of Polish‐speaking children aged 2.97–6.15 years (*M* = 4.79, SD = 0.84), with 64 items for production. Despite these differences, both studies highlighted poor agreement at an individual level. Our study ran an exploratory analysis on agreement on seven items that were both part of the CDI checklist as well as the CLT‐SK direct assessment. Our results based on 30 child–parent dyads also indicated an overestimation of the assessment of children's vocabulary by parents (see Table 5). For example, 16 out of 30 responses were in agreement between parent and child response for the item *pomaranč* (an orange), with 12 parents indicating that the child says the word and correctly produced the item in the test, and 4 parents indicated that the child does NOT say the word and the child did NOT produce the item in the test. However, 14 out of 30 parents overestimated their children's knowledge, such that parents indicated that the child says the word, but the child failed to correctly name the item. Our study, conducted in yet another language and community (Slovak), echoed the findings of previous research, highlighting parents’ tendency to overestimate children's knowledge. However, the picture was more complex when further characteristics of the items were considered, as discussed in the following section.

As we had a few items, we considered the factors that may have influenced the differences between agreement on the items without analysing them statistically. Specifically, we considered the acquisition and item trajectories based on a large dataset of CDI data in Slovak as reported by Frank et al. (2017) via *Wordbank*. Using the analysis of growth curves for individual words on a CDI form, we checked the production at 30th months. Of the seven words, three words showed perfect or excellent agreement between parents’ judgement and child performance: Wordbank data confirmed that the item *auto* (car) is spoken by more than 98% of children in the 30th month, and the word *pit* (drink) is spoken by more than 86%. In both cases, parents’ judgement almost perfectly matches their children's performance. On the other hand, the word *ananas* (pineapple) was shown to be spoken by only about half (55%) of the children in their 30th month. This item shows the smallest agreement between parents' judgement and child performance, with parents reporting the production of the word, but children able to name it correctly much less frequently. This also applies to the other items we included, such as *lepiť /lepidlo* (to glue/glue), *voňať* (smell) and *pomaranč* (orange), with 61%, 69% and 78% children producing those in their 30th month, according to Wordbank.

Together, those figures indicate a trend that later acquired words show a lower level of agreement between parents’ judgement and child performance. One item that behaved differently by this measure is *plakať* (cry), which had a good agreement (27 out of 30 agreed). The item is produced by 76% of children in their 30th month, so lower than what we saw with the high agreement items. However, this item features heavily in Slovak child–directed speech and was reported among the most frequent verbs (Brestovičová 2018). In summary, it appears that early acquisition and very high frequency of individual items in the input contribute to better agreement between parents’ judgement and child's direct assessment of their lexical skills. This is in line with findings from Bennetts et al. (2016), who found that agreement was generally better for children with very weak or very strong language skills and poorer for children with average language skills. Similarly, Łuniewska et al. (2024) reported agreement declining alongside an increase in vocabulary size. They also echo Łuniewska et al. (2024) word of warning that clinicians and/or researchers should be cautious and not make assumptions about the acquisition of individual items. These findings at the individual‐item level should be interpreted with caution, given the small number of items analysed and the limited generalisability of item‐level patterns; they nonetheless provide valuable preliminary insights to guide future research.

### 4.1 Limitations and Future Research

Although our study brings novel insights, caution is needed when interpreting and generalising the results. The information about participants’ demographic background was not evaluated, and recent studies highlighted that factors such as race, education and gender present potential biases in evaluating early vocabulary (Kachergis et al. 2023). Future research should increase the size and diversity of the sample and collect background information to re‐evaluate the relationship and assess the impact of demographic factors.

The present study did not examine the relationship between CLT scores and a more direct measure of expressive vocabulary, such as NDW derived from spontaneous or narrative‐elicited language samples. Pilot work in this area, including an unpublished Master's thesis (Valečíková 2016), found a significant positive association between NDW in free‐play samples and CLT‐SK scores in 2‐year‐old children. This suggests that the link between spontaneous language use and vocabulary knowledge assessed via direct tasks emerges early. Future research could extend this investigation to older children. However, MAIN narratives may not be optimal for this purpose, as some of the children's stories in our study were shorter than 1 min, which is a length generally considered the minimum for reliable lexical diversity estimates (Heilmann et al. 2010). Longer language samples, elicited through alternative narrative tasks or free play, are likely to yield more robust and reliable NDW measurements.

This study only included a sample of TD children and established that the concurrent relationship between lexical measures holds in typical development. Kapalková and Slančová (2017) showed that children with DLD as a group showed weaker lexical skills as measured by CLT‐SK. In future studies, the relationships between various lexical metrics should also be assessed in clinical populations, in particular in children with DLD. While expressive vocabulary might not be the strongest marker of DLD on an individual level, as other factors impact vocabulary size beyond the DLD status, it appears to be informative about a child's progress and prognosis. Therefore, it is important to have a valid and reliable tool, which would allow for tracking changes in expressive vocabulary.

### 4.2 Conclusions

Having a reliable and valid tool for assessing vocabulary is a vital step for identifying and monitoring children who might need support with their language. Our findings show that all tasks and metrics used as indicators of lexical skills were significantly positively related, and this provides support for convergent validity for CLT‐SK. Importantly, the task is quick, child‐friendly, and the administration‐associated costs are low. In addition, the task has an electronic as well as paper version, reducing the need for a hard copy for every clinician/practice. From the outset, the task has also been developed for diverse populations (Haman et al. 2015; Haman et al. 2017), and CLT principles have been applied across many language versions of the task. The current study contributes to the growing body of knowledge on CLT by providing further evidence on the convergent validity of CLT‐SK and highlights the mutual relationships between different lexical assessments. The findings from our study reflect the complex interplay between different types of vocabulary and the cognitive and social factors that influence lexical development.

## Conflicts of Interest

The authors declare no conflicts of interest.

## Data Availability

The raw/processed data required to reproduce the above findings cannot be shared at this time due to legal/ethical reasons.
